# Effect of Lead on Human Middle Ear Epithelial Cells

**DOI:** 10.1155/2018/5058729

**Published:** 2018-03-06

**Authors:** Shin Hye Kim, Sun Hwa Shin, Yoon Young Go, Sung-Won Chae, Jae-Jun Song

**Affiliations:** Department of Otorhinolaryngology-Head and Neck Surgery, Korea University Medical Center, Korea University College of Medicine, Seoul, Republic of Korea

## Abstract

Lead is a ubiquitous metal in the environment, but no studies have examined lead toxicity on the middle ear. Here, we investigated lead toxicity and its mechanism in human middle ear epithelial cells (HMEECs). Moreover, we investigated the protective effects of amniotic membrane extract (AME) and chorionic membrane extract (CME) against lead toxicity in HMEECs. Cell viability was analyzed using the cell counting kit, and reactive oxygen species (ROS) activity was measured using a cellular ROS detection kit. After lead(II) acetate trihydrate treatment, mRNA levels of various genes were assessed by semiquantitative real-time polymerase chain reaction. Following treatment with AME or CME after lead exposure, the changes in cell viability, ROS activity, and gene expression were analyzed. Exposure to >100 *μ*g/mL of lead(II) acetate trihydrate caused a significant decrease in cell viability and increased ROS production in HMEECs. Lead exposure significantly increased the mRNA expression of genes encoding inflammatory cytokines and mucins. Administration of AME or CME restored cell viability, reduced ROS activity, and ameliorated mRNA levels. Our findings suggest that environmental lead exposure is related to the development of otitis media, and AME and CME may have antioxidative and anti-inflammatory effects against lead toxicity.

## 1. Introduction

Otitis media (OM) is a group of inflammatory diseases of the middle ear. The presence of inflammatory cytokines in middle ear fluid samples obtained from children with OM has been reported [1, 2]. OM is a common inflammatory disease among children, and more than 50% of children experience one or more episodes of OM by the age of three years [3]. Fluid and mucus trapped in the middle ear by OM may lead to conductive hearing loss and delays in speech development and cognitive abilities [4]. Thus, the identification and control of potentially preventable risk factors for OM, such as air pollution exposure, have significant implications for children's healthcare.

Lead is a common and versatile metal that is widely distributed in the environment, leading to human exposure. However, lead exposure through environmental (canned food, water pipes, soil, paint, plastics, household dust, air, etc.) or occupational routes can cause lead poisoning [5]. Particularly, in developing countries, lead is present in particulate form in the air and can be inhaled along with other heavy metals [6].

The major mechanism of lead toxicity is thought to be increased oxidative stress [7]. Lead induces an imbalance between the production of free radicals and detoxification of reactive intermediates or repair of the resulting damage. Oxidative stress occurs through two simultaneous pathways: generation of reactive oxygen species (ROS) and depletion of antioxidant reserves [8]. Ionic mechanisms and apoptosis have also been suggested as mechanisms of lead toxicity [9]. Lead can be substituted for other bivalent cations such as Ca^2+^, Mg^2+^, and Fe^2+^ as well as monovalent cations such as Na^+^, affecting various fundamental physiological processes [9]. Although lead toxicity does not occur through a single unifying mechanism, its ability to substitute for Ca^2+^ is a common factor in its toxicity [10]. Lead has been reported to induce activation of several cellular and molecular processes, such as apoptosis in cancer cell models and rats [11, 12].

Lead toxicity via ROS generation, ionic mechanism, and apoptosis has been demonstrated using* in vitro* and* in vivo* experimental models. However, no studies have examined the effects of this heavy metal on the middle ear. Therefore, this study evaluated the effects and mechanism of lead toxicity on human middle ear epithelial cells (HMEECs). In a previous study, we showed that amniotic membrane extract (AME) and chorionic membrane extract (CME) have anti-inflammatory effects on HMEECs; therefore, we evaluated the protective effect of AME and CME on lead toxicity [13].

## 2. Materials and Methods

### 2.1. Cell Culture

HMEECs (kindly provided by Dr. David J. Lim, House Ear Institutes, Los Angeles, CA, USA) established using human papillomavirus E6/E7 genes for the study of normal cell biology and pathological processes associated with development of OM were used. HMEECs were maintained in a mixture of Dulbecco's modified Eagle's medium (Invitrogen, Carlsbad, CA, USA) and bronchial epithelial basal medium (Lonza, Basel, Switzerland) (1 : 1) [14] and kept in an incubator with a humidified atmosphere at 37°C containing 95% air and 5% CO_2_. The growth medium was changed every third or fourth day. The doubling time of HMEECs is approximately 3 days, and cells were used for subsequent studies after 6 days. After approximately 1 week, the cells were stimulated with 10, 50, or 100 *μ*g/mL lead(II) acetate trihydrate (Sigma, St. Louis, MO, USA) suspended in phosphate-buffered saline (PBS) for 24 h. As a control group, HMEECs were treated with only PBS without lead(II) acetate trihydrate.

### 2.2. Cell Viability Assay

To analyze the viability of HMEECs, a cell viability assay was performed using a cell counting kit (CCK-8, Dojindo Laboratories, Kumamoto, Japan). HMEECs were seeded in 96-well plates, with each well containing 1 × 10^4^ cells. The cells were treated with 0, 6.25, 12.5, 25, 50, 100, 200, 300, 400, 500, 600, 700, or 800 *μ*g/mL of lead(II) acetate trihydrate on the following day. The cells were washed twice with PBS, and 10% CCK-8 solution was added to each well after 24 h. The plates were incubated for 2 h at 37°C, and the contents of the plates were mixed at room temperature (20–25°C) for 5 min using a shaker. The optical density was measured at 450 nm using a microplate reader (Spectra Max plus 384, Molecular Devices, Sunnyvale, CA, USA). The results were obtained from three repeated experiments using triplicate samples.

### 2.3. Cell Apoptosis Assay

After exposure to lead(II) acetate trihydrate for 24 h, apoptotic cells were detected in real-time using a caspase-3 detection kit (NucView™ 488 Caspase-3, Cat Number 4440, Biotium, Inc., Fremont, CA, USA). Following cell seeding, plates were warmed to 37°C for 30 min and then scanned using an IncuCyte Zoom system (Essen BioScience, Ann Arbor, MI, USA).

### 2.4. ROS Activity Assays

HMEECs (1.5 × 10^4^) were treated with lead(II) acetate trihydrate for 24 h. ROS activity in the cells was quantified using a 2′,7′-dichlorofluorescin diacetate (DCFDA) cellular ROS detection assay kit (Abcam, Cambridge, UK). Briefly, the cells were washed twice with PBS and then incubated with 100 *μ*L DCFDA in culture medium at 37°C for 30 min. The cells were washed twice with PBS and analyzed using an IncuCyte Zoom system (Essen Bioscience). As a positive control to assess the ROS activity, 50 *μ*g/mL tert-butyl hydrogen peroxide (TBHP) was used.

### 2.5. Real-Time Reverse Transcriptase Polymerase Chain Reaction

*TNF-α* and* COX-2* are inflammatory cytokines genes related to the OM [15].* MUC5AC* and* MUC5B* are mucins genes, and they reflect the mucins production and OM induction [16].* ENaC-α*,* ENaC-β*, and* ENaC-γ* are genes encoding epithelial sodium channels and they reflect the mucociliary transport in the middle ear mucosa [17].* AQP-4* is gene of aquaporin in middle ear mucosa and it reflects the homeostasis of the middle ear cavity [18]. To quantify the expression of* TNF-α*,* COX-2*,* MUC5AC*,* MUC5B*,* ENaC-α*,* ENaC-β*,* ENaC-γ*, and* AQP-4* in HMEECs, real-time polymerase chain reaction (RT-PCR) was performed. After exposure to lead, RNA was extracted from HMEECs using TRIzol, and reverse transcription was performed using a cDNA synthesis kit (Takara Bio, Inc., Shiga, Japan).

RT-PCR was performed using an ABI Prism 7300 real-time PCR system (Applied Biosystems, Foster City, CA, USA). Each reaction mixture contained 10 *μ*L of LightCycler 480SYBR Green I Master (Roche, Mannheim, Germany), 1 *μ*L of cDNA, and 5 pmol each of sense and antisense primer in a final volume of 20 *μ*L. Reaction mixtures were incubated at 95°C for 5 min to activate the FastStart Taq DNA Polymerase. This was followed by amplification for 45 cycles (one cycle: 15 s at 95°C, 30 s at 60°C, and 30 s at 72°C). The data were analyzed using LightCycler 480 software 1.5 (Roche). Results were obtained from three repeated experiments using triplicate samples.

### 2.6. Effect of Amniotic Membrane Extract and Chorionic Membrane Extract on Lead Exposure

Human amniotic and chorionic membranes are known to have anti-inflammatory effects [19]. Amniotic and chorionic membranes were obtained from the placenta of a pregnant woman who delivered at our tertiary center. The study was approved by the institutional review board of Korea University Guro Hospital (KUGH14239-002), and all participants provided written informed consent. The amniotic and chorionic membranes were washed with PBS. After homogenization and sonication of the membranes, AME and CME were obtained from the supernatant via three rounds of centrifugation at 13000 rpm for 30 min. To examine the protective effect of AME and CME on lead toxicity, the changes in cell viability, ROS activity, and gene expression after simultaneous treatment with AME and CME in lead-treated HMEECs were analyzed.

### 2.7. Statistical Analysis

All data are expressed as means ± standard deviations. Statistical analyses were performed using SPSS ver. 12.0 (SPSS, Inc., Chicago, IL, USA). One-way analysis of variance (ANOVA) was used to determine significant differences between the control and experimental groups at each time or dose point. When significant differences were identified by ANOVA, Scheffe's *F*-test was used to correct for multiple comparisons. A *p* value < 0.05 was considered to indicate a statistically significant difference.

## 3. Results

### 3.1. Lead Altered the Morphology and Induced Apoptosis of HMEECs

**Figure 1:**
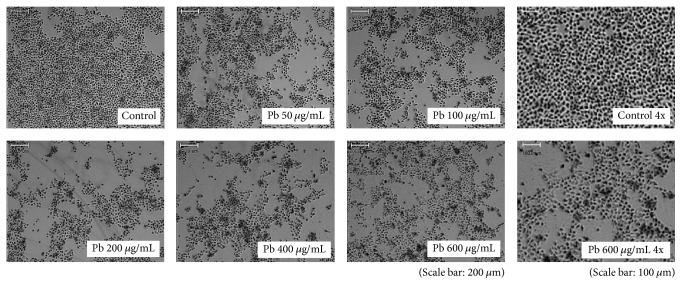
Cell morphology of human middle ear epithelial cells (HMEECs) following exposure to lead for 24 h. As a control group, HMEECs were treated with only PBS without lead(II) acetate trihydrate: administration of lead(II) acetate trihydrate resulted in reduced cell size and cell detachment from the Petri dishes.

**Figure 2:**
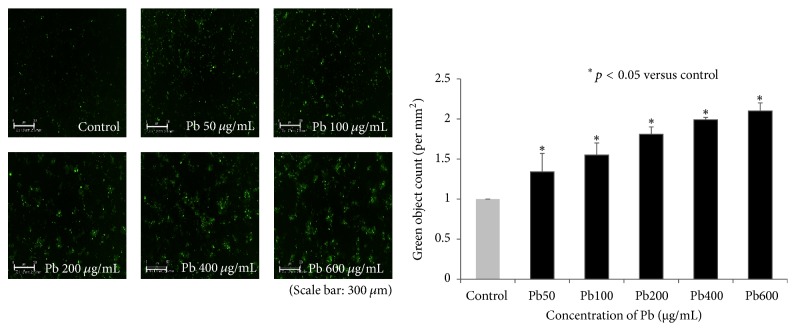
Apoptosis of human middle ear epithelial cells (HMEECs) following exposure to lead for 24 h. Apoptotic cells were detected by fluorescence using an IncuCyte Zoom system: the numbers of apoptotic cells increased with increasing concentration of lead(II) acetate trihydrate. Error bars indicate the standard error of the mean (SEM).

The morphology of HMEECs was altered following exposure to lead(II) acetate trihydrate (Figure 1). The nuclei of control cells were round and homogeneously stained. Addition of lead(II) acetate trihydrate resulted in reduced cell size and cell detachment from the Petri dishes. Apoptotic cells were detected by fluorescence, and the numbers of apoptotic cells increased with increasing concentrations of the lead(II) acetate trihydrate (Figure 2).

### 3.2. Lead Reduced the Cell Viability of HMEECs

**Figure 3:**
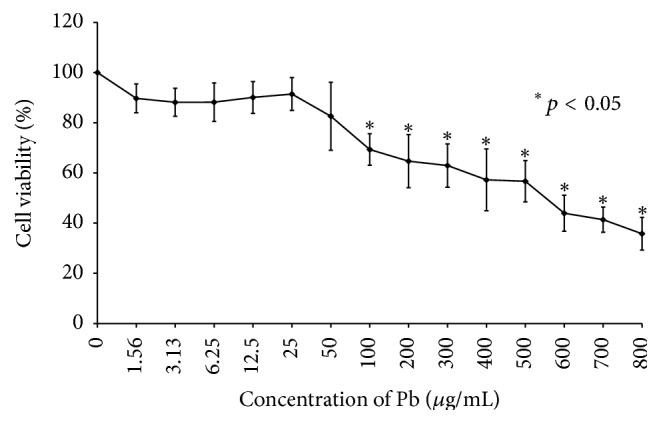
Cell viability of human middle ear epithelial cells (HMEECs) following exposure to lead: exposure to more than 100 *μ*g/mL of lead(II) acetate trihydrate for 24 h significantly decreased HMEECs viability as compared to control cells. Error bars indicate the standard error of the mean (SEM).

Cell viability assays (CCK-8) showed that exposure to more than 100 *μ*M of lead(II) acetate trihydrate for 24 h significantly decreased HMEECs viability compared to control cells (Figure 3).

### 3.3. Lead Increased ROS Production in HMEECs

**Figure 4:**
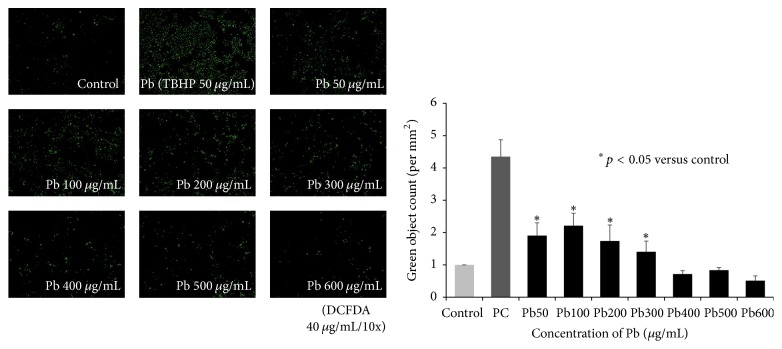
Reactive oxygen species (ROS) activity of human middle ear epithelial cells (HMEECs) following exposure to lead. As a positive control, 50 *μ*g/mL tert-butyl hydrogen peroxide (TBHP) was used: ROS activity was increased in cells exposed to 50–300 *μ*g/mL of lead(II) acetate trihydrate compared to control cells, but the ROS activity was decreased in cells exposed to >400 *μ*g/mL lead(II) acetate trihydrate compared to control cells. Error bars indicate the standard error of the mean (SEM).

Increased ROS activity was detected in cells exposed to 50–300 *μ*g/mL of lead(II) acetate trihydrate compared to control cells (Figure 4). However, ROS activity was decreased in cells exposed to >400 *μ*g/mL lead(II) acetate trihydrate compared to control cells, perhaps because of cell death caused by the high concentration of lead.

### 3.4. Lead Altered Gene Expression in HMEECs

**Figure 5:**
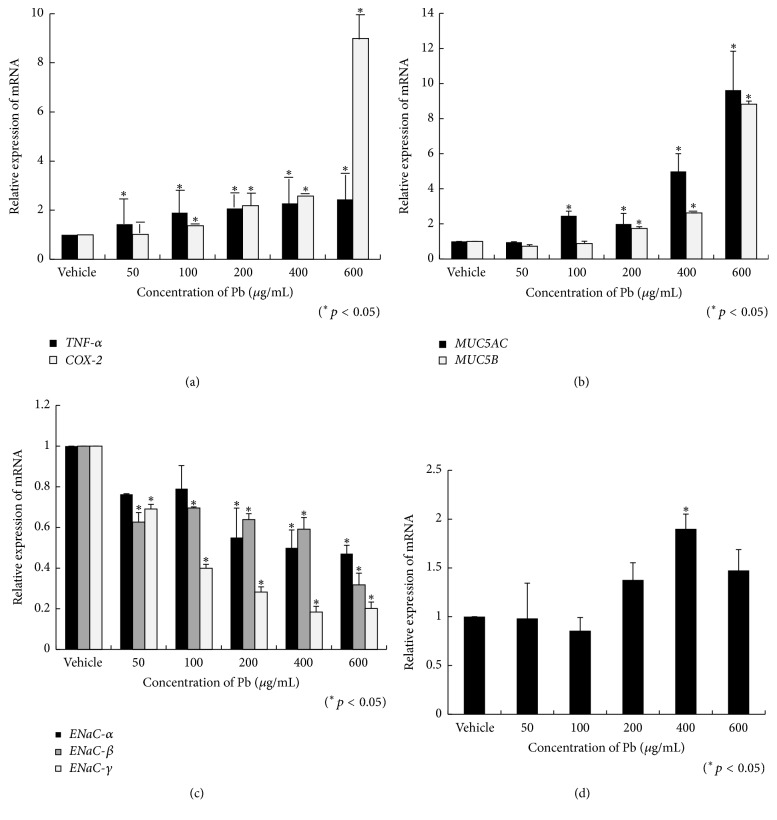
Alteration of gene expression in human middle ear epithelial cells (HMEECs) following exposure to lead: lead(II) acetate trihydrate concentrations of >50 and >100 *μ*g/mL for 24 h significantly increased gene expression of* TNF-α* and* COX-2* HMEECs, respectively (a). Lead(II) acetate trihydrate at >100 and >200 *μ*g/mL for 24 h significantly increased gene expression of* MUC5AC* and* MUC5B* in HMEECs, respectively (b). Lead(II) acetate trihydrate at >200, >50, and >50 *μ*g/mL for 24 h significantly decreased gene expression of* ENaC-α*,* ENaC-β*, and* ENaC-γ*, respectively (c). Lead(II) acetate trihydrate at 400 *μ*g/mL for 24 h significantly increased gene expression of* AQP-4* (d). Error bars indicate the standard error of the mean (SEM).

When cells were stimulated with >50 and >100 *μ*g/mL lead(II) acetate trihydrate for 24 h, gene expression of* TNF-α* and* COX-2* was significantly increased in HMEECs, respectively (*p* < 0.05, Figure 5(a)). Gene expression of* MUC5AC* and* MUC5B* in HMEECs significantly increased following stimulation with >100 and >200 *μ*g/mL of lead(II) acetate trihydrate for 24 h, respectively (*p* < 0.05, Figure 5(b)). Gene expression of* ENaC-α*,* ENaC-β*, and* ENaC-γ* significantly decreased when cells were stimulated with >200, >50, and >50 *μ*g/mL of lead(II) acetate trihydrate for 24 h, respectively (*p* < 0.05, Figure 5(c)). Gene expression of* AQP-4* significantly increased when cells were stimulated with 400 *μ*g/mL lead(II) acetate trihydrate for 24 h (*p* < 0.05, Figure 5(d)).

### 3.5. Amniotic and Chorionic Membrane Extracts Reduced Lead Toxicity

**Figure 6:**
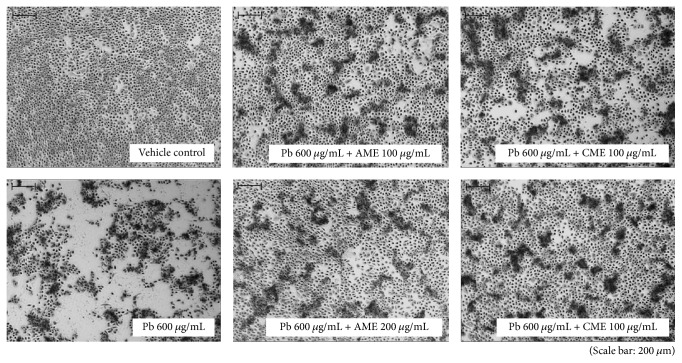
Effect of amniotic membrane extract (AME) and chorionic membrane extract (CME) on cell morphology in human middle ear epithelial cells (HMEECs) following exposure to lead for 24 h: reduced cell size and condensed nuclei by exposure to 600 *μ*g/mL lead(II) acetate trihydrate was recovered by treatment with AME or CME.

**Figure 7:**
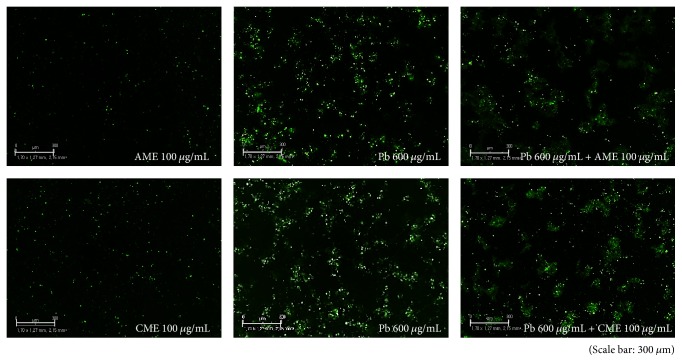
Effect of amniotic membrane extract (AME) and chorionic membrane extract (CME) on apoptosis in human middle ear epithelial cells (HMEECs) following exposure to lead for 24 h: addition of 100 *μ*g/mL AME or CME to cells treated with 600 *μ*g/mL lead(II) acetate trihydrate decreased the number of apoptotic cells.

**Figure 8:**
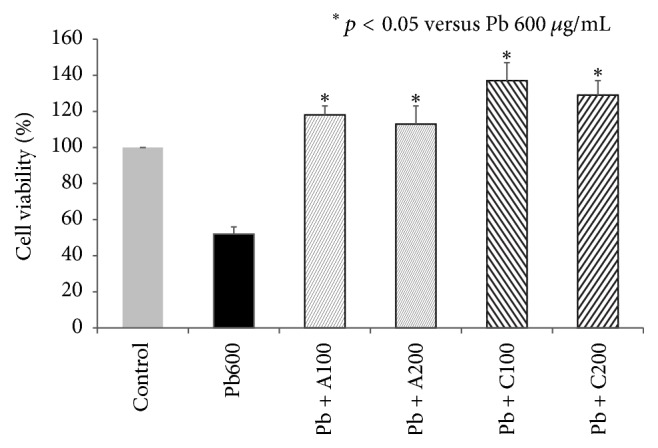
Effect of amniotic membrane extract (AME) and chorionic membrane extract (CME) on cell viability of human middle ear epithelial cells (HMEECs) following exposure to lead for 24 h: administration of 100 and 200 *μ*g/mL AME or CME significantly increased cell viability of HMEECs exposed to 600 *μ*g/mL lead(II) acetate trihydrate. Error bars indicate the standard error of the mean (SEM).

**Figure 9:**
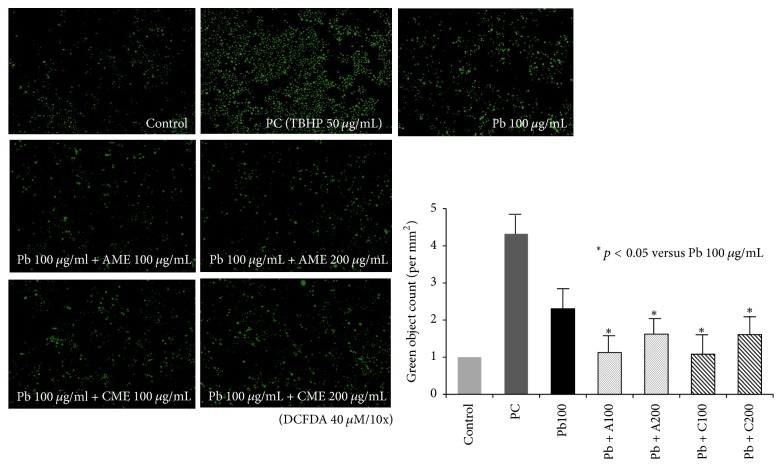
Effect of amniotic membrane extract (AME) and chorionic membrane extract (CME) on reactive oxygen species (ROS) activity of human middle ear epithelial cells (HMEECs) following exposure to lead. As a positive control, 50 *μ*g/mL tert-butyl hydrogen peroxide (TBHP) was used: administration of 100 and 200 *μ*g/mL AME or CME significantly decreased ROS activity in HMEECs exposed to 100 *μ*g/mL lead(II) acetate trihydrate. Error bars indicate the standard error of the mean (SEM).

HMEECs were decreased in size, and nuclei were condensed following exposure to 600 *μ*g/mL lead(II) acetate trihydrate; however, the morphology was recovered by treatment with AME or CME (Figure 6). Addition of AME or CME to 600 *μ*g/mL lead(II) acetate trihydrate also decreased the number of apoptotic cells (Figure 7). The number of HMEECs was significantly reduced by 600 *μ*g/mL lead(II) acetate trihydrate; however, the negative effect of lead on the cell viability of HMEECs was significantly reduced by administration of 100 and 200 *μ*g/mL AME or CME (Figure 8). ROS activity was increased by administration of 600 *μ*g/mL lead(II) acetate trihydrate; however, administration of 100 and 200 *μ*g/mL AME or CME decreased ROS activity (Figure 9).

### 3.6. Amniotic and Chorionic Membrane Extracts Recovered Normal Gene Expression in HMEECs following Exposure to Lead

**Figure 10:**
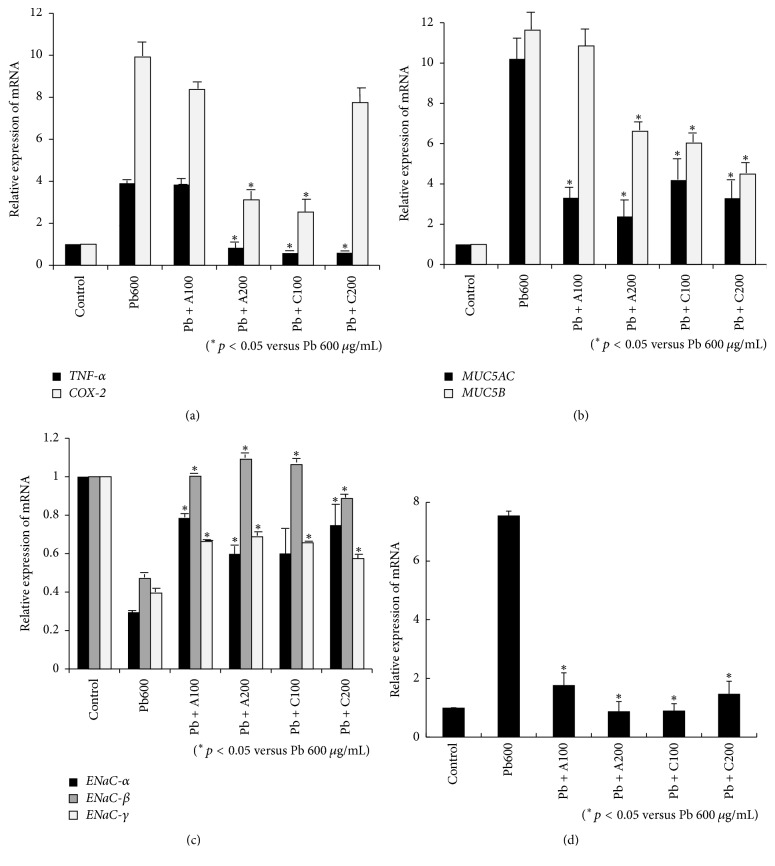
Effect of amniotic membrane extract (AME) and chorionic membrane extract (CME) on alterations of gene expression in human middle ear epithelial cells (HMEECs) following exposure to lead: administration of AME and CME decreased gene expression of* TNF-α* and* COX-2* (a),* MUC5AC*,* MUC5B* (b), and* AQP-4* (d) in HMEECs exposed to 600 *μ*g/mL lead(II) acetate trihydrate. Administration of AME and CME increased gene expression of* ENaC-α*,* ENaC-β*, and* ENaC-γ* (c) in HMEECs exposed to 600 *μ*g/mL lead(II) acetate trihydrate. Error bars indicate the standard error of the mean (SEM).

The increased expression of* TNF-α* and* COX-2* following administration of 600 *μ*g/mL lead(II) acetate trihydrate was decreased following administration of AME and CME (Figure 10(a)). The increased expression of* MUC5AC* and* MUC5B* following exposure to 600 *μ*g/mL lead(II) acetate trihydrate was also decreased following administration of AME and CME (Figure 10(b)). The decreased expression of* ENaC-α*,* ENaC-β*, and* ENaC-γ* following 600 *μ*g/mL lead(II) acetate trihydrate administration was also recovered by AME and CME (Figure 10(c)). The increased expression of* AQP-4* following 600 *μ*g/mL lead(II) acetate trihydrate administration was decreased following administration of AME and CME (Figure 10(d)).

## 4. Discussion

Lead is an insidiously hazardous material that has the potential to cause irreversible negative health effects. Lead is known to interfere with numerous physiological functions, primarily affecting the central nervous, hematopoietic, hepatic, and renal systems [7, 20]. Lead is present in the form of airborne particulate matter, along with other heavy metals. We predicted that lead, similar to other air pollutants or smoke, can enter the middle ear space through the Eustachian tubes or via the systemic circulation. The potential for lead exposure to have adverse effects is heightened in children for three reasons: young children often place objects in their mouths, resulting in ingestion of dust and soil; intake of lead per unit of body weight is higher for children than for adults; and young children are undergoing rapid development and are consequently more vulnerable than adults to lead toxicity [21–23].

Lead persists in the environment as it is a nonbiodegradable material. Environmental exposure to lead became relatively high in the latter part of the 20th century, and concerted efforts were required to reduce lead exposure. In the late 1970s, the median blood lead level of US preschool children was 15 *μ*g/dL, and 88% of children had a level exceeding 10 *μ*g/dL [24]. Based on the National Health and Nutrition Examination Survey (NHANES) (1991–1994, USA), the mean blood lead level among American children aged 1–5 years was 2.7 *μ*g/dL, and 4.4% of these children (890,000 in the USA population) had elevated blood lead levels [25]. In humans with chronic exposure of lead containing substances, immune, reproductive, and cardiovascular systems are adversely affected at blood levels of 10 *μ*g/dL and even lower [26]. Children stratified into the low-lead group (lead < 10 *μ*g/dL) were reported to have a lower relative risk of respiratory illnesses and OM than those with lead ≥ 10 *μ*g/dL [27].

Studies on lead toxicity have reported that the presence of lead in the body induces toxicological manifestations through various cellular, intracellular, and molecular mechanisms. Oxidative stress has been reported as a major mechanism of lead toxicity. Under the influence of lead, the onset of oxidative stress occurs via two distinct but simultaneously operating pathways: generation of ROS, such as hydroperoxides (HO2^•^) and singlet oxygen and hydrogen peroxide (H_2_O_2_), and depletion of antioxidant reserves [14]. The ionic mechanism of lead toxicity mainly operates because lead can substitute for other bivalent cations such as Ca^2+^, Mg^2+^, and Fe^2+^ as well as monovalent cations such as Na^+^ (although bivalent cations are more readily substituted), affecting various fundamental physiological processes [19].

To investigate the mechanism of lead toxicity, considering the HMEECs with short-term (24 h) exposure of lead(II) acetate trihydrate, this* in vitro* study used higher concentration of lead (e.g., 100 *μ*g/mL: 1000 times for 10 *μ*g/dL,* in vivo* criteria). We demonstrated that lead decreased the cell viability of HMEECs and increased ROS production. Lead also induced inflammatory mucins production. Inflammatory cytokines and mucins secretion play important roles in the development of OM. Inflammatory cytokines, including COX-2, TNF-*α*, NF-*κ*B, IL-1, IL-6, and IL-8, play a critical role in the initiation of mucosal changes, inflammatory response in the middle ear, and mucins secretion [15, 16]. Increased expression of genes encoding inflammatory cytokines and mucins has been detected in cigarette smoke-, diesel-, cadmium-, and Asian sand dust-induced OM [28–31]. In the present study, lead caused increased gene expression of inflammatory cytokines (*TNF-α* and* COX-2*) and mucins (*MUC5AC* and* MUC5B*). Lead reduced the expression of genes encoding for epithelial sodium channels (*ENaC-α*,* ENaC-β*, and* ENaC-γ*) and consequently deteriorated mucociliary transport in the middle ear mucosa. Lead also induced gene expression of aquaporin* (AQP-4)* in the middle ear mucosa and consequently dysregulated the homeostasis of the middle ear cavity. Later,* in vivo* study confirming the concentration of lead inducing the toxicity will be needed.

The amniotic and chorionic membranes, located in the inner side of the placenta and formed by cubical cells and an inner mesodermal tissue, have been shown to have antiapoptotic, antiangiogenic, and anti-inflammatory effects on epithelial cells [32, 33]. The mesenchymal stem cells obtained from mesoderm of human amniotic membrane possess immunosuppressive functions through soluble factors such as prostanoids and proteins [34]. AME, an extract of the human amniotic membrane, has also been shown to have anti-inflammatory effects. From the experiment using human corneal epithelial cells, homogenized human AME less than 3 kDa had a greater capacity to decrease the inflammation and secretion of IL-6 and IL-8 [35]. Human AME has been proven to contain human neutrophil peptides, lysozyme, LL-37 (C-terminal part of the only human cathelicidin), bactericidal/permeability-increasing protein, calprotectin, and ubiquitin, and to show anti-inflammatory effects [36, 37]. In the present study, both AME and CME showed antiapoptotic and anti-inflammatory effects on HMEECs following exposure to lead. The first clinical use of AME was to treat epithelial defects of the cornea [38], and it is used for the treatment of ocular and dermatologic injuries and diseases. Despite the fact that therapies with AME have been used to ameliorate acute and chronic inflammatory diseases of eye and skin, the precise mechanisms by which these cells or soluble factors exert their function are yet poorly understood. Additional experiments to confirm what components of AME and CME show antiapoptotic and anti-inflammatory effects will be needed.

## 5. Conclusions

Our study revealed a causal relationship between environmental lead exposure and OM. One limitation of this* in vitro* study is that we evaluated lead toxicity in the cell model of the human middle ear. In the future,* in vivo* studies and clinical trials are needed to confirm the toxicity of lead and protective effects of AME and CME in the middle ear.
