# Corrigendum to “Effect of Lead on Human Middle Ear Epithelial Cells”

**DOI:** 10.1155/2018/3252078

**Published:** 2018-08-09

**Authors:** Shin Hye Kim, Sun Hwa Shin, Yoon Young Go, Sung-Won Chae, Jae-Jun Song

**Affiliations:** ^1^Department of Otorhinolaryngology-Head and Neck Surgery, Korea University Medical Center, Korea University College of Medicine, Seoul, Republic of Korea; ^2^Department of Otorhinolaryngology-Head and Neck Surgery, Haeundae Paik Hospital, Inje University College of Medicine, Busan, Republic of Korea

In the article titled “Effect of Lead on Human Middle Ear Epithelial Cells” [1], there was a missing affiliation for the first author. The corrected authors' list and affiliations are shown above.
